# Chinese Yam Polysaccharide Alleviates DSS-Induced Ulcerative Colitis After Antibiotic Pretreatment

**DOI:** 10.3390/foods15101633

**Published:** 2026-05-08

**Authors:** Yushun Qian, Fuhao Leng, Yan Yu, Yi Wu, Jiaxin Zhang, Lanlan Cheng, Mingyue Shen, Jianhua Xie

**Affiliations:** State Key Laboratory of Food Science and Resources, Nanchang University, No. 235 Nanjing East Road, Nanchang 330047, China; ssskjkszpj@163.com (Y.Q.); 407900240012@email.ncu.edu.cn (F.L.); camille1234567891@163.com (Y.Y.); ah_wuyi@163.com (Y.W.); m15551390587@163.com (J.Z.); 17721833632@163.com (L.C.)

**Keywords:** Chinese yam polysaccharide, microbiota-depleted, intestinal barrier, hepatic oxidative stress, NF-κB and MAPK pathways, metabolic pathways

## Abstract

This study investigated whether the therapeutic efficacy of Chinese yam polysaccharide (CYP) against ulcerative colitis (UC) depends on an intact gut microbiota. A dextran sulfate sodium (DSS)-induced colitis mouse model was established, and one treatment group received broad-spectrum antibiotics (ABXs) before CYP administration to deplete the intestinal microbiota. CYP markedly attenuated colonic injury, reduced disease activity, and suppressed inflammatory mediators under both microbiota-intact and microbiota-depleted conditions. CYP also enhanced intestinal barrier integrity, as evidenced by reduced serum endotoxin levels and increased expression of MUC-2, Claudin-1, Occludin, and ZO-1. In addition, CYP improved hepatic antioxidant status by increasing GSH-Px and catalase activities and decreasing malondialdehyde levels. Moreover, CYP reduced the activation of the NF-κB and MAPK signaling pathways, with similar trends observed under microbiota-depleted conditions. Microbiota profiling showed that CYP partially corrected DSS-induced dysbiosis, whereas the ABX + CYP group exhibited distinct microbial patterns with enrichment of carbohydrate-related metabolic pathways predicted by PICRUSt2. Collectively, these findings suggest that CYP retains protective efficacy after antibiotic pretreatment, indicating that its effects may not be exclusively dependent on gut microbiota modulation, possibly involving direct actions on immune and intestinal epithelial cells.

## 1. Introduction

Ulcerative colitis (UC), a chronic and relapsing subtype of inflammatory bowel disease (IBD), predominantly affects the colon and is characterized by diffuse mucosal inflammation [1]. The worldwide incidence and prevalence of UC have risen steadily, especially in newly industrialized regions, imposing substantial clinical and economic burdens on healthcare systems [2]. Although the underlying cause of UC remains elusive, it is generally attributed to a multifactorial interaction among genetic susceptibility, environmental influences, immune dysregulation, and gut microbiota dysbiosis [3,4,5]. Clinically, UC manifests as bloody diarrhea, abdominal pain, and tenesmus [6]. The disease typically follows an unpredictable course with alternating periods of remission and relapse, profoundly compromising patients’ quality of life. Current therapeutic strategies mainly focus on inducing and maintaining clinical remission through pharmacological interventions such as 5-aminosalicylic acid, corticosteroids, immunomodulators, and biologics [7]. However, many patients experience severe adverse effects, including corticosteroid dependence or resistance, underscoring the urgent need for novel therapeutic targets and more effective treatment approaches [8].

Modulation of the gut microbiota has arisen as a pivotal strategy in understanding UC pathophysiology and developing therapeutic interventions. Patients with ulcerative colitis frequently exhibit dysbiosis, manifested as diminished microbial diversity and a decreased presence of beneficial short-chain fatty acid (SCFA)-producing microorganisms [9]. This microbial imbalance disrupts intestinal barrier integrity, exacerbates mucosal inflammation, and promotes aberrant activation of immune responses [10]. Consequently, restoring a balanced gut microbiota has attracted growing attention as a therapeutic strategy for UC. In this context, natural polysaccharides—complex carbohydrates derived from plants, fungi, algae, and microorganisms—have attracted increasing interest because of their prebiotic potential and multifunctional bioactivities [11]. These polysaccharides exhibit excellent safety profiles and can modulate multiple biological pathways, rendering them promising candidates for UC intervention. Several studies have demonstrated that natural polysaccharides, such as pectin-derived oligosaccharides and oat β-glucans, alleviate experimental colitis primarily through microbiota-dependent mechanisms [12,13]. However, in severe colitis, chronic inflammation extensively damages the intestinal mucosal barrier [14]. This disruption not only amplifies local immune responses but also destroys the ecological niche essential for commensal microbiota survival. Likewise, recurrent antibiotic exposure can drive the microbial ecosystem into a dysbiotic and therapeutically recalcitrant state, hindering subsequent restoration [15]. Under these conditions, strategies relying solely on microbiota modulation may be inadequate. Emerging evidence suggests that some natural polysaccharides can directly exert anti-inflammatory effects and ameliorate colitis independent of microbiota modulation [16,17]. For instance, *Polygonatum cyrtonema*-derived polysaccharides have been shown to ameliorate colitis by modulating inflammatory responses and enhancing epithelial barrier function, independent of the gut microbiota [18]. Such microbiota-independent mechanisms render these polysaccharides compelling therapeutic candidates for patients with impaired microbial homeostasis. Therefore, polysaccharides capable of exerting direct host-protective effects may represent advantageous therapeutic options.

Chinese yam polysaccharide (CYP), a bioactive macromolecule extracted from Chinese yam, has been reported to alleviate colitis by modulating intestinal inflammation and gut microbiota composition [19,20]. In our previous study, we found that CYP ameliorated colitis by modulating the gut microbiota, and that this effect was reproducible following fecal microbiota transplantation. However, whether the ameliorative effects of CYP on UC are dependent on gut microbiota modulation remains unclear. Previous studies have reported that pretreatment with broad-spectrum antibiotics alone does not significantly affect DSS-induced UC mice, in terms of colon length, intestinal barrier protein expression, or levels of inflammatory cytokines [21,22]. Therefore, this study aimed to systematically investigate the ameliorative effects of CYP in a DSS-induced UC mouse model. A microbiota-depletion model was employed to determine whether CYP retained protective efficacy under conditions of profound microbiota disruption. Furthermore, the molecular mechanisms underlying CYP’s protective effects were explored, with particular focus on intestinal barrier integrity, inflammatory mediators, and signaling pathways. This work provides new insights into host-directed effects of natural polysaccharides, suggesting that their actions may not be entirely dependent on gut microbiota modulation.

## 2. Materials and Methods

### 2.1. Materials

In our previous study, CYP was isolated and structurally characterized. In the present study, CYP was prepared following the same extraction and purification procedures [23]. Enzyme-linked immunosorbent assay (ELISA) kits for interleukin-10 (IL-10), IL-6, IL-1β, tumor necrosis factor-alpha (TNF-α), and myeloperoxidase (MPO) were purchased from Wuhan Boster Biological Technology Co., Ltd. (Wuhan, China). Assay kits for catalase (CAT, S0051), malondialdehyde (MDA, S0131M), and glutathione peroxidase (GSH-Px, S0057S) and the BCA protein assay kit (P0011) were acquired from Beyotime Biotechnology (Shanghai, China). Vancomycin, neomycin sulfate, metronidazole, and ampicillin were obtained from Shanghai Macklin Biochemical Technology Co., Ltd. (Shanghai, China). Primary antibodies against p38 mitogen-activated protein kinase (p38), phospho-p38 (p-p38), extracellular signal-regulated kinase (ERK), p-ERK, c-Jun N-terminal kinase (JNK), p-JNK, p65, p-p65, NF-kappa-B inhibitor alpha (IκBα), and p-IκBα were purchased from Cell Signaling Technology (Boston, MA, USA). The antibody against β-actin was purchased from Beijing Zhongshan Golden Bridge Biotechnology Co., Ltd. (Beijing, China).

### 2.2. Animal Experiment

Forty male specific pathogen-free C57BL/6J mice (6 weeks old, 18–20 g) were acquired from Beijing Vital River Laboratory Animal Technology Co., Ltd. (Beijing, China). The mice underwent acclimatization for one week under controlled conditions (12 h light/dark cycle, 20–25 °C, 65% relative humidity) with free access to food and water. Following acclimation, forty mice were randomly assigned to four groups: control, DSS, CYP, and ABX + CYP. From days 7 to 14, mice in the CYP group received CYP solution (200 mg/kg body weight) via oral gavage. The selection of this dose was supported by previous studies showing that 200 mg/kg CYP effectively alleviates ulcerative colitis in mice and exhibits strong immunomodulatory activity [23,24]. Meanwhile, mice in the ABX + CYP group received a combination of broad-spectrum antibiotics (1 mg/mL metronidazole, 1 mg/mL neomycin sulfate, 1 mg/mL vancomycin, and 1 mg/mL ampicillin) by gavage. Mice in the control and DSS groups received an equivalent volume of normal saline via gavage. From days 14 to 21, mice in the CYP and ABX + CYP groups received CYP solution by oral gavage, whereas mice in the control and DSS groups continued to receive normal saline. During the final 7 days of the experimental period, all mice except those in the control group were administered drinking water supplemented with 3% (*w*/*v*) DSS. Blood samples were obtained 24 h following the final treatment via retro-orbital puncture, and the mice were subsequently sacrificed via cervical dislocation. Collected tissues were promptly frozen and maintained at −80 °C until further research. All animal procedures complied with the ethical guidelines approved by the Institutional Animal Care and Use Committee (IACUC) of Nanchang University (Nanchang, China).

### 2.3. Body Weight and Disease Activity Index

Body weight of the mice was recorded daily to monitor changes during the experimental period. Stool consistency and presence of blood were observed and recorded daily, and the disease activity index (DAI) was calculated according to the criteria presented in Table S1.

### 2.4. Histopathological Analysis

Following fixation in 4% paraformaldehyde, colonic tissues were paraffin-embedded and sectioned for histological examination. Paraffin sections were deparaffinized, rehydrated, and treated with a staining pretreatment solution for 1 min. The tissue sections were subjected to hematoxylin staining for 3–5 min, rinsed, dehydrated in 95% ethanol for 1 min, and subsequently counterstained with eosin for 15 s. Subsequently, sections underwent sequential dehydration in absolute ethanol, n-butanol, and xylene before being mounted with neutral resin. Stained sections were observed under a Leica Aperio LV1 digital pathology scanner (Leica Microsystems, Wetzlar, Germany). Nuclei appeared blue, whereas cytoplasm was stained pink to red. Histopathological scores were assigned based on the extent of lesions, crypt damage, depth of invasion, and inflammatory severity, as described in Table S2. The cumulative score derived from these parameters was used to calculate the final histopathological index.

### 2.5. Immunofluorescence Assay

Paraffin-embedded tissue sections were processed through deparaffinization, rehydration, and subsequent antigen retrieval, followed by blocking with 5% bovine serum albumin for 30 min. The sections were subjected to overnight incubation at 4 °C with primary antibodies against MUC-2, ZO-1, Occludin, and Claudin-1. After washing three times with PBS (5 min each), the slides were subjected to incubation with the corresponding fluorescent secondary antibody for 50 min under dark conditions. After another three PBS washes, the slides were incubated with DAPI solution for 10 min in the dark. Subsequently, the slides were washed three additional times with PBS and treated with autofluorescence quenching agent B for 5 min. The slides were rinsed with distilled water for 10 min, followed by further washing under running water. Fluorescence images were obtained using a fluorescence microscopy system and processed by Wuhan Servicebio Technology Co., Ltd. (Wuhan, China).

### 2.6. Determination of Antioxidants and Cytokines in Liver and Intestinal Tissue

Colon tissues were processed in pre-cooled PBS (1:10, *w*/*v*) and homogenized using a mechanical homogenizer (Servicebio, Wuhan, China) at 70 Hz for 60 s, repeated for three cycles with 30 s intervals for cooling. Homogenized samples were centrifuged at 12,000× *g* for 10 min at 4 °C, after which the supernatants were harvested for subsequent analyses. The concentrations of cytokines (IL-10, IL-1β, IL-6, and TNF-α) and MPO activity were measured using commercial ELISA kits in accordance with the manufacturer’s protocol. For the assessment of hepatic oxidative stress, liver tissues were processed following the manufacturer’s descriptions for the corresponding assay kits. The activities of CAT and GSH-Px, along with MDA levels, were quantified in liver homogenates. In addition, total protein levels in liver supernatant samples were quantified using a BCA protein assay kit.

### 2.7. Western Blotting Assay

Total proteins from colon tissues were extracted using pre-cooled immunoprecipitation (IP) lysis buffer containing 1 mM phenylmethylsulfonyl fluoride (PMSF) protease inhibitor. The protein lysates were resolved by sodium dodecyl sulfate–polyacrylamide gel electrophoresis on 7.5–12.5% gradient gels and transferred onto polyvinylidene difluoride membranes via a wet transfer method. After blocking with 5% nonfat milk in TBST for 50 min at ambient temperature, membranes were subjected to overnight incubation at 4 °C with primary antibodies (1:1000–1:2000 in TBST). Following three successive washes with TBST (10 min each), the membranes were incubated with horseradish peroxidase (HRP)-conjugated secondary antibodies (1:5000 dilution) for 50 min. Protein bands were visualized using enhanced chemiluminescence substrate and quantified using the Gel Doc XR+ imaging system (Bio-Rad, Hercules, CA, USA).

### 2.8. 16S rRNA Gene Sequencing

Genomic DNA from fecal microbiota was extracted using the MagBeads FastDNA Kit for Soil (MP Biomedicals, Irvine, CA, USA). The V3-V4 hypervariable regions of 16S rRNA genes were subjected to amplification using primer pairs 806R (5′-GGACTACHVGGGGTWTCTAAT-3′) and 338F (5′-ACTCCTACGGGAGGCAGCA-3′). Amplification fragments were purified and subsequently analyzed by paired-end sequencing (2 × 250 bp) using the Illumina NovaSeq PE250 sequencing platform (Illumina, San Diego, CA, USA). Operational Taxonomic Unit (OTU) clustering was performed using Vsearch (v2.13.4), followed by α- and β-diversity analyses in QIIME2 (v2024.5). PICRUSt2 was employed to predict the metabolic functional potential of the microbial community inferred from 16S rRNA gene sequences using the MetaCyc (https://metacyc.org/; accessed on 20 June 2025), KEGG (https://www.kegg.jp/; accessed on 20 June 2025), and COG (https://www.ncbi.nlm.nih.gov/COG/; accessed on 20 June 2025) databases.

### 2.9. Statistical Analysis

All data are presented as mean ± standard deviation, and statistical analyses were performed using GraphPad Prism 9.5.0 software (GraphPad Software, San Diego, CA, USA). Differences among groups were analyzed using one-way analysis of variance (ANOVA) and subsequent multiple comparisons were conducted using Dunnett’s post hoc test. * *p* < 0.05, ** *p* < 0.01, *** *p* < 0.001, and **** *p* < 0.0001 indicate significant differences between groups.

## 3. Results

### 3.1. CYP Improved Colonic Pathology and Inflammatory Response in Colitis Mice Under Microbiota-Depleted Conditions

**Figure 1:**
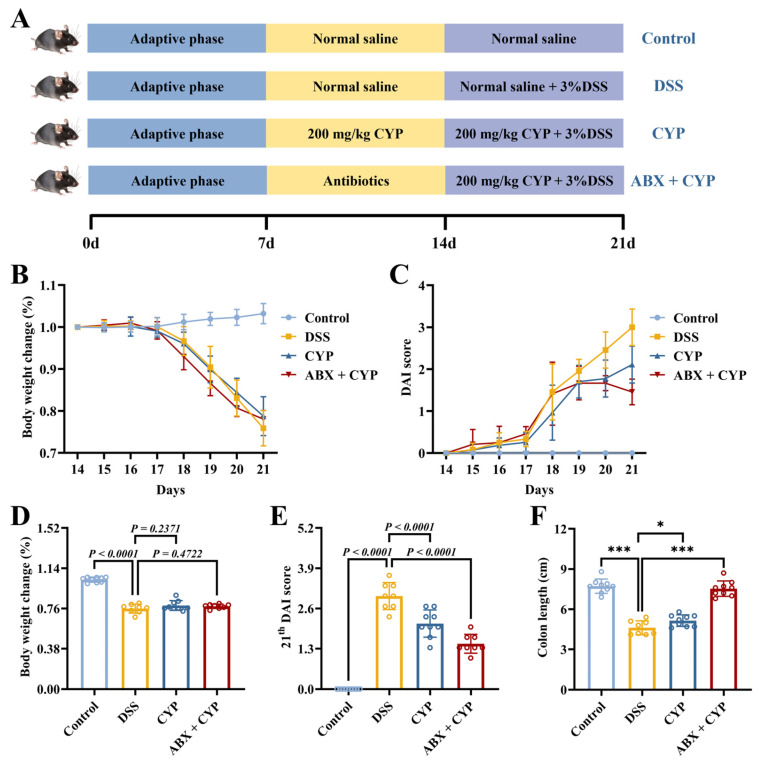
Effects of CYP and ABX + CYP supplementation on DSS-induced colitis symptoms. (**A**) Schematic diagram of the experimental protocol. (**B**) Body weight change rate. (**C**) Disease activity index (DAI) scores. (**D**) Body weight change rate on day 21. (**E**) DAI score on day 21. (**F**) Colon length. * *p* < 0.05 and *** *p* < 0.001 indicate significant differences between groups.

To determine whether the ameliorative effects of CYP on UC were mediated by the gut microbiota, an animal experiment was designed as illustrated in Figure 1A. Prior to DSS induction (3%, *w*/*v*), mice in the ABX + CYP group were pretreated with a broad-spectrum antibiotic cocktail to deplete the gut microbiota. The therapeutic efficacy of CYP, with or without antibiotic pretreatment, in DSS-induced colitis was evaluated by measuring body weight, DAI scores, and colon length. As shown in Figure 1B,D, the DSS group exhibited a significant reduction in body weight relative to the control group (*p* < 0.0001). Both CYP and ABX + CYP treatments did not significantly attenuate body weight loss compared with the DSS group. According to the DAI results (Figure 1C,E), the DSS group showed markedly increased disease severity relative to the control group (*p* < 0.0001). CYP and ABX + CYP administration attenuated the DSS-induced increase in DAI scores, both showing significantly lower scores than the DSS group on day 21 (*p* < 0.0001). Moreover, colon length was markedly reduced in the DSS group relative to the control group (Figure 1F, *p* < 0.0001). Colon length in the CYP group showed a marked increase compared with that in the DSS group (*p* < 0.05), whereas the ABX + CYP group exhibited a more pronounced increase relative to the DSS group (*p* < 0.001). These findings indicate that CYP alleviated DSS-induced colon shortening even under microbiota-depleted conditions. Collectively, these findings suggest that CYP mitigated disease severity in DSS-induced colitis despite profound microbiota depletion.

### 3.2. CYP Ameliorated Inflammation and Enhanced Hepatic Antioxidant Capacity in Colitis Mice Under Microbiota-Depleted Conditions

**Figure 2:**
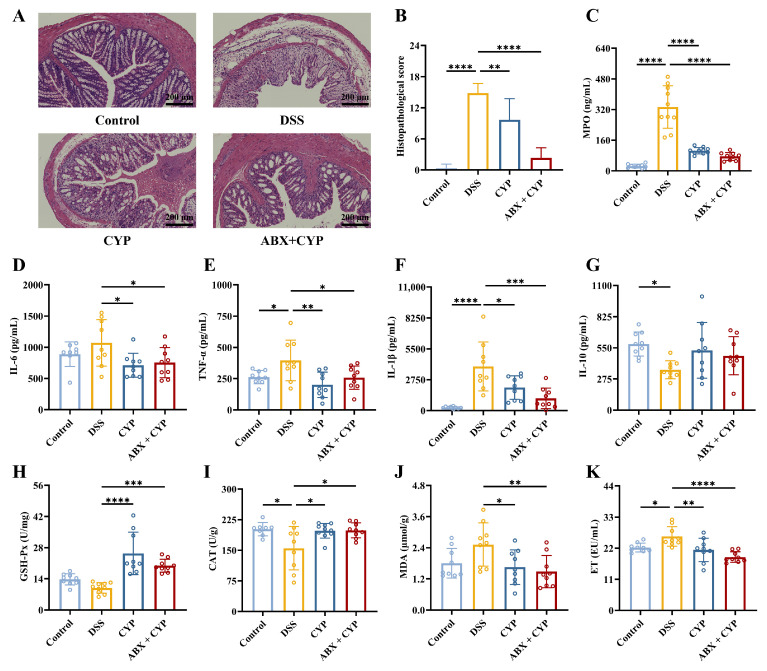
Effects of CYP and ABX + CYP on colonic injury, inflammation, and hepatic antioxidant capacity. (**A**) Representative H&E-stained images of colonic sections from each group. (**B**) Quantitative histological scoring. (**C**) MPO activity in colonic tissue. (**D**–**G**) Concentrations of IL-6, TNF-α, IL-1β, and IL-10 in colonic tissue. (**H**,**I**) Activities of GSH-Px and CAT in hepatic tissue. (**J**) Hepatic MDA content. (**K**) Serum endotoxin (ET) levels. * *p* < 0.05, ** *p* < 0.01, *** *p* < 0.001, and **** *p* < 0.0001 indicate significant differences between groups.

Subsequently, histopathological analysis of colon tissues was performed to evaluate mucosal injury. Hematoxylin and eosin staining revealed that the DSS group exhibited severe mucosal injury characterized by epithelial disruption, goblet cell depletion, extensive inflammatory infiltration, and ulcer formation compared with the control group (Figure 2A,B). In contrast, both CYP and ABX + CYP treatments significantly ameliorated histological damage, as indicated by reduced lesion areas, restoration of crypt architecture, and attenuation of inflammatory infiltration. To further characterize the inflammatory response, key inflammatory mediators in colonic tissues were quantified. DSS markedly increased MPO activity and levels of TNF-α and IL-1β while reducing IL-10 levels relative to the control group (Figure 2C–G, *p* < 0.05). CYP treatment markedly reduced MPO and pro-inflammatory cytokine levels (*p* < 0.05). Similarly, the ABX + CYP group showed significant reductions in inflammatory mediators relative to the DSS group. These findings demonstrate that CYP alleviated colonic inflammation and tissue injury under conditions of antibiotic-induced microbiota depletion.

In addition to colonic injury, DSS exposure frequently induces hepatic oxidative stress [25]. In this study, hepatic antioxidant capacity was evaluated by measuring the activities of GSH-Px and CAT, along with MDA content (Figure 2H–J). DSS did not significantly alter GSH-Px activity relative to the control group. However, both CYP and ABX + CYP treatment markedly increased enzymatic activity compared with the DSS group. DSS treatment significantly decreased CAT activity relative to the control group, while both CYP and ABX + CYP treatment significantly restored CAT activity relative to the DSS group. As shown in Figure 2J, MDA content exhibited a non-significant increase in the DSS group relative to the control group. Both CYP and ABX + CYP treatments significantly reduced MDA content relative to the DSS group. These findings indicate CYP treatment improved antioxidant enzyme activities and reduced lipid peroxidation, even under microbiota-depleted conditions.

### 3.3. CYP Restored Intestinal Barrier Function in Colitis Mice Under Microbiota-Depleted Conditions

**Figure 3:**
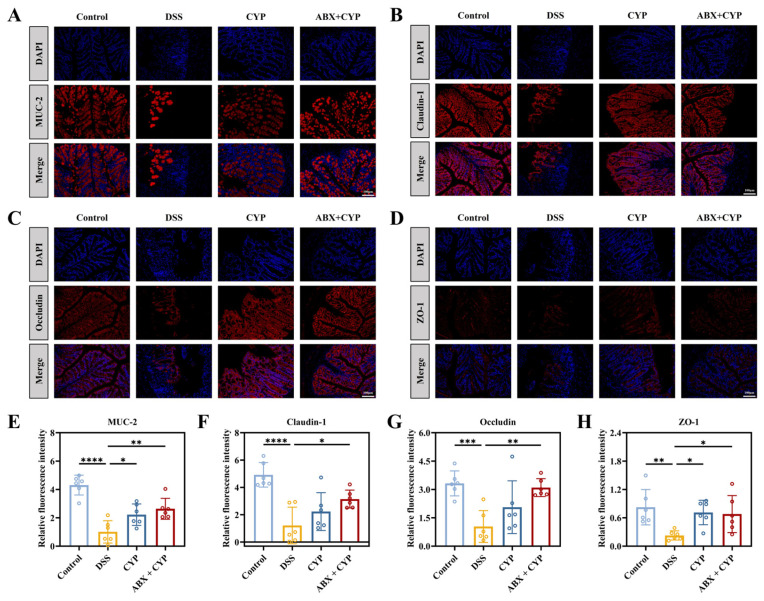
Effects of CYP and ABX + CYP on intestinal barrier function. (**A**–**D**) Immunofluorescence staining of MUC-2, Claudin-1, Occludin, and ZO-1 in colonic tissue. Red fluorescence indicates the target protein, blue fluorescence indicates the nucleus, and “Merge” represents the merged image. (**E**–**H**) Quantification of relative fluorescence intensity (×10^7^) of MUC-2, Claudin-1, Occludin, and ZO-1. * *p* < 0.05, ** *p* < 0.01, *** *p* < 0.001, and **** *p* < 0.0001 indicate significant differences between groups.

Impaired intestinal barrier function is an essential contributor to the pathogenesis of colitis [26]. To evaluate barrier integrity, serum endotoxin (ET) levels were measured. The DSS group showed significantly elevated ET concentrations relative to the control group (*p* < 0.05), suggesting impaired intestinal barrier function (Figure 2K). Both CYP and ABX + CYP treatments significantly reduced serum ET levels compared with the DSS group, suggesting reduced ET translocation into the circulation. Furthermore, immunofluorescence analysis was performed to examine the fluorescence intensity of MUC-2, a major mucin component of the colonic mucus layer, and the tight junction proteins (Claudin-1, Occludin, and ZO-1). Fluorescence imaging (Figure 3A–D) and quantitative analysis (Figure 3E–H) revealed that the expression of MUC-2, Claudin-1, Occludin, and ZO-1 was markedly reduced in the DSS group relative to the control group (*p* < 0.01). By contrast, CYP administration significantly increased the expression levels of intestinal barrier-related proteins, particularly MUC-2 and ZO-1 (*p* < 0.05). Importantly, the ABX + CYP group exhibited significant upregulation of all analyzed proteins. These findings indicate that CYP restored intestinal barrier integrity and reduced permeability by enhancing the expression of MUC-2, ZO-1, Occludin, and Claudin-1. Moreover, this protective effect was achieved under conditions of microbiota depletion.

### 3.4. CYP Suppressed MAPK and NF-κB Signaling Activation in DSS-Induced Colitis Under Microbiota-Depleted Conditions

**Figure 4:**
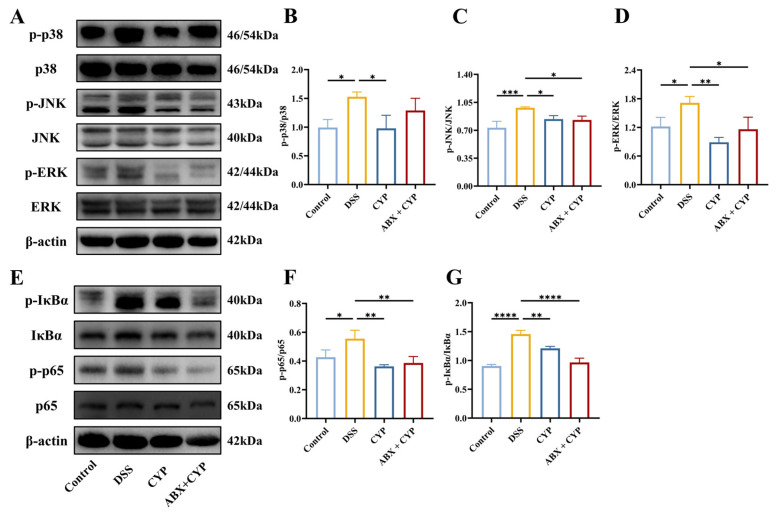
Effect of CYP and ABX + CYP on NF-κB and MAPK signaling pathways. (**A**) Representative Western blot images of p-p38, p38, p-JNK, JNK, p-ERK, and ERK. (**B**–**D**) Gray-scale analysis of the corresponding proteins. (**E**) Representative Western blot images of p-IκBα, IκBα, p-p65, and p65. (**F**,**G**) Gray-scale analysis of the corresponding proteins. β-actin served as the loading control. (*n* = 3). * *p* < 0.05, ** *p* < 0.01, *** *p* < 0.001, and **** *p* < 0.0001 indicate significant differences between groups.

The MAPK and NF-κB signaling pathways are central regulators of inflammatory responses in UC [27]. To investigate whether the protective effects of CYP against colonic inflammation were associated with changes in NF-κB and MAPK signaling after antibiotic pretreatment, Western blot analysis of key proteins in these pathways was performed. Relative to the control group, the DSS group exhibited significant activation of the MAPK pathway, as indicated by increased phosphorylation of ERK, JNK, and p38 (*p* < 0.05, Figure 4A–D). This suggested that DSS-induced colitis was associated with activation of MAPK signaling. Treatment with CYP alone significantly reduced the phosphorylation levels of ERK, JNK, and p38 relative to the DSS group. Notably, the ABX + CYP group similarly suppressed JNK and ERK phosphorylation relative to the DSS group (*p* < 0.05). In addition, we assessed the activation status of the NF-κB pathway by measuring p-IκBα and p-p65 levels (Figure 4E–G). Relative to the control group, the DSS group exhibited increased phosphorylation of both IκBα and p65, indicating stimulation of the NF-κB pathway. CYP treatment significantly reduced p-IκBα and p-p65 levels relative to the DSS group, suggesting that the anti-inflammatory effects of CYP may be associated with reduced NF-κB pathway activation. Similarly, compared with the DSS group, the ABX + CYP group showed decreased NF-κB activation, with reduced levels of both p-IκBα and p-p65 (*p* < 0.01). Together, these results suggest that CYP-mediated protection may be associated with reduced activation of key inflammatory pathways, and this effect was still observed after antibiotic pretreatment.

### 3.5. Gut Microbiota Structure and Composition in Colitis Mice

**Figure 5:**
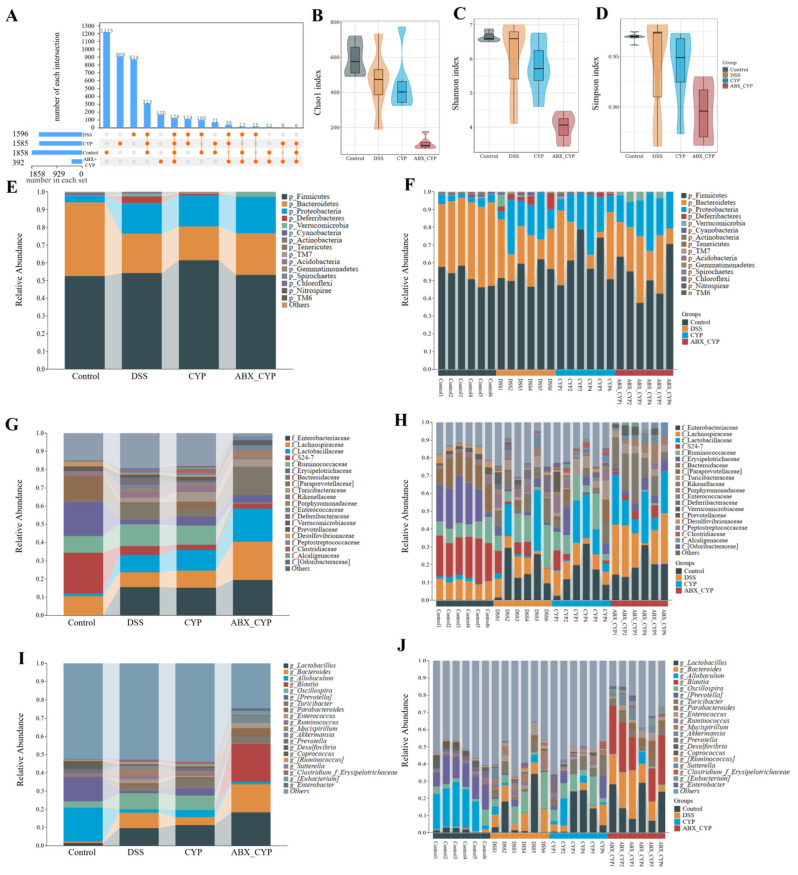
Effects of CYP and ABX + CYP on gut microbiota diversity and taxonomic composition. (**A**) UpSet plot among different groups. Orange filled circles indicate the groups included in each intersection, and orange connecting lines indicate the intersections among the corresponding groups. (**B**–**D**) Alpha diversity indices of gut microbiota, including the Chao1 richness index (**B**), Shannon diversity index (**C**), and Simpson diversity index (**D**). (**E**–**J**) Relative abundance of the top 20 bacterial taxa at the phylum (**E**,**F**), family (**G**,**H**), and genus (**I**,**J**) levels.

The gut microbiota composition in mice was characterized using 16S rRNA gene sequencing. As shown in the UpSet plot (Figure 5A), the control, DSS, CYP, and ABX + CYP group exhibited 1858, 1596, 1585, and 392 operational taxonomic units (OTUs), respectively, with 1215, 874, 909, and 170 OTUs unique to each group. Relative to the control group, DSS administration alone caused a reduction in the total number of OTUs. The ABX + CYP group showed the lowest OTU count, whereas CYP treatment alone did not significantly affect the OTU number. This suggests that CYP itself did not cause a marked reduction in OTU counts. In addition, the α-diversity indices, including Chao1, Shannon, and Simpson, were all markedly reduced in the ABX + CYP group (Figure 5B–D). Previous studies have shown that reductions in OTU counts and α-diversity indices can be used to evaluate effective depletion of major gut microbiota in mice after antibiotic treatment [28,29]. Therefore, the marked decreases in OTU counts and α-diversity indices observed in the present study suggest that antibiotic pretreatment effectively removed most of the gut microbiota in mice.

Community composition bar plots revealed distinct microbial profiles across multiple taxonomic ranks, including phylum, family, and genus (Figure 5E–J). At the phylum classification level, Firmicutes and Bacteroidetes were predominant in all groups. DSS treatment altered the relative abundance of major phyla relative to the control group. Although antibiotic pretreatment in the ABX + CYP group markedly reduced microbial richness and diversity, Firmicutes and Bacteroidetes remained detectable among the predominant phyla in the residual microbiota. At the family level, DSS treatment induced compositional shifts characterized by altered abundances of several inflammation-associated families, including Enterobacteriaceae and Enterococcaceae. Similarly, at the genus level, DSS treatment reshaped the microbial community composition, while CYP intervention partially adjusted the relative abundance of several dominant genera. By contrast, the ABX + CYP group displayed a markedly simplified microbial structure with reduced diversity compared with the other groups.

**Figure 6:**
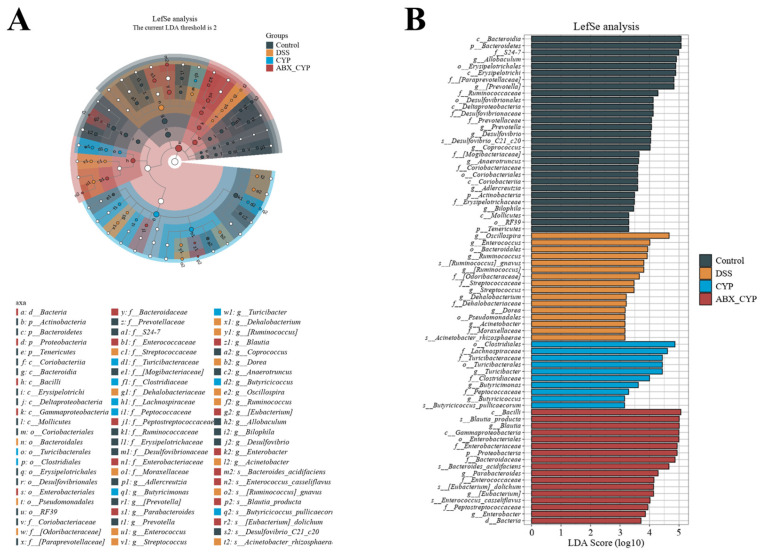
LEfSe analysis of differential gut microbiota among groups. (**A**) Cladogram illustrating phylogenetic distribution of significantly enriched taxa. (**B**) Linear discriminant analysis (LDA) score histogram of differentially abundant taxa identified by LEfSe analysis.

LEfSe analysis was conducted to identify differentially abundant bacterial taxa, characterize differences in microbial community structure among groups, and detect statistically significant biomarkers (Figure 6A,B). The LDA score histogram displayed the discriminative taxa enriched in each group. These results indicated that the predominant taxa in the control group were *c_Bacteroidia* and *p_Bacteroidetes*; DSS group was *g_Oscillospira*; CYP group were *o_Clostridiales* and *f_Lachnospiraceae*; and ABX + CYP group were *c_Bacilli*, *s_Blautia_producta*, *g_Blautia, c_Gammaproteobacteria* and *o_Enterobacteriales*. These findings show that DSS administration substantially disrupted the gut microbiota, whereas CYP treatment remodeled the community and enriched taxa associated with intestinal homeostasis, including butyrate-producing members of the family Lachnospiraceae. By contrast, following antibiotic pretreatment, the microbial community in the ABX + CYP group exhibited features of dysbiosis.

### 3.6. Predicted Functional Tendencies of the Residual Gut Microbiota in ABX + CYP Group Mice

**Figure 7:**
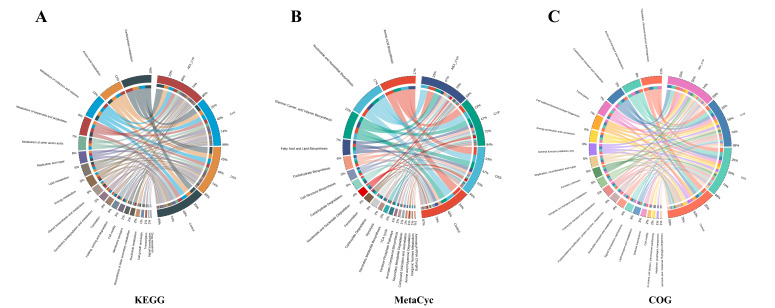
Functional pathway prediction based on PICRUSt2 analysis. Chord diagrams of (**A**) KEGG, (**B**) MetaCyc and (**C**) COG pathways.

The functional potential of the gut microbiota was predicted using PICRUSt2 with reference to the KEGG, MetaCyc, and COG databases. As illustrated in the chord diagram (Figure 7), distinct predicted metabolic tendencies were observed among the different treatment groups. Comparative analysis revealed that the ABX + CYP group exhibited enrichment of predicted carbohydrate-related pathways across all three databases (Figure S2). No comparable enrichment was observed in the control, DSS, or CYP groups. Specifically, KEGG analysis showed that the ABX + CYP group was mainly associated with predicted enrichment of carbohydrate metabolism and biosynthesis of other secondary metabolites pathways (Figure S2A and Figure 7A). Similarly, MetaCyc pathway analysis revealed predicted enrichment of pathways involved in carbohydrate degradation and secondary metabolite degradation in the ABX + CYP group (Figure S2B and Figure 7B). Furthermore, COG functional classification showed a predicted increase in carbohydrate transport and metabolism pathways in the ABX + CYP group (Figure S2C and Figure 7C). These results suggest that the residual microbial community in the ABX + CYP group may have a distinct predicted functional tendency, with a bias toward carbohydrate metabolism and secondary metabolite processing.

## 4. Discussion

In this work, the potential therapeutic effects of CYP were investigated in an acute colitis murine model, with a particular spotlight on whether its effects were dependent on an intact gut microbiota. Using antibiotic-induced microbiota depletion, we found that CYP retained marked protective effects against DSS-induced colitis even under microbiota-suppressed conditions. Both CYP and ABX + CYP treatments alleviated disease severity, attenuated colonic injury, restored intestinal barrier integrity, and enhanced hepatic antioxidant capacity. In addition, these treatments modulated key inflammatory signaling pathways, collectively contributing to their protective efficacy against DSS-induced colitis.

As an approach to disrupt the gut microbiota, antibiotics are widely used in microbiome research to induce pseudo-germ-free conditions in animals. This study was designed based on previous reports suggesting that pretreatment with broad-spectrum antibiotics alone does not markedly affect either normal mice or DSS-induced UC mice [21,22]. In several similar studies, meaningful and scientifically valid results have been obtained even in the absence of an ABX + DSS group [28,30]. Accordingly, an ABX + DSS group was not included in the present experimental design, which is a limitation of this study. In the present study, both CYP and ABX + CYP treatments did not significantly attenuate body weight loss compared with the DSS group. Similar changes in body weight have also been observed in a previous study using LPS-induced acute inflammatory mouse models [31]. This may be partly attributed to a lagged effect in body weight recovery. Body weight restoration is a relatively slow physiological process that depends on the recovery of appetite, nutrient absorption, and metabolic homeostasis. CYP improved clinical and histological indices of colitis even under microbiota-depleted conditions. This observation suggests that CYP-mediated protection does not rely exclusively on microbial regulation and may involve direct modulation of host immune responses. Similar host-directed effects have been reported for other polysaccharides, including those derived from *Polygonatum sibiricum* [18]. Consistent with these reports, both CYP and ABX + CYP reduced pro-inflammatory cytokines (IL-6, TNF-α, IL-1β), suggesting that the anti-inflammatory effects of polysaccharides may not be strictly dependent on microbiota modulation.

Oxidative stress plays a pivotal role in the development of UC, contributing to both intestinal inflammation and systemic damage, particularly in the liver. During inflammatory tissue injury, excessive production of reactive oxygen species disrupts the hepatic antioxidant defense system, leading to pronounced oxidative damage [32]. The impaired antioxidant defense further exacerbates inflammation, establishing a vicious cycle between oxidative injury and inflammatory response [33,34]. Several studies have demonstrated that natural polysaccharides, such as those derived from *Morchella esculenta* and seaweed, can mitigate oxidative damage by upregulating antioxidant enzyme activities, including GSH-Px and CAT, while reducing lipid peroxidation products such as MDA [35,36]. The present findings corroborated these reports, showing that CYP treatment improved hepatic antioxidant capacity in colitis mice by increasing GSH-Px and CAT activities while decreasing MDA levels. Notably, these effects were evident even in the ABX + CYP group, indicating that the antioxidant effects of CYP were preserved after antibiotic-induced microbiota depletion.

The preservation of intestinal integrity is essential for maintaining gastrointestinal homeostasis. Intestinal barrier dysfunction, a hallmark of colitis pathogenesis, is characterized by impaired tight junction integrity and increased epithelial permeability [37,38]. Compromised barrier integrity promotes the translocation of luminal antigens across the epithelium into the lamina propria, where they subsequently trigger immune cells and downstream inflammatory cascades [39]. This persistent inflammatory milieu hampers mucosal repair, forming a self-perpetuating cycle of barrier dysfunction and chronic inflammation that drives UC progression and exacerbation [40]. Previous studies have shown that polysaccharides, including those derived from *Astragalus membranaceus* and *Panax ginseng*, can restore intestinal barrier integrity by enhancing mucin secretion and upregulating tight junction protein expression [41,42]. Consistent with these findings, CYP and ABX + CYP treatments effectively restored the expression and proper localization of critical mucosal protective factors, including MUC-2, ZO-1, Occludin, and Claudin-1, thereby reinforcing epithelial integrity and reducing endotoxin translocation. Unlike many prebiotic therapies that rely on microbiota modulation to enhance barrier integrity, CYP improved the expression of tight junction proteins and mucin after antibiotic-induced microbiota depletion, supporting its potential utility in settings where microbial homeostasis is disrupted.

The NF-κB and MAPK signaling cascades play critical roles in the development of UC by orchestrating complex inflammatory responses [43,44]. Upon IKK-mediated phosphorylation and degradation of IκBα, NF-κB is activated and translocates p65 into the nucleus, thereby upregulating the expression of pro-inflammatory genes [45]. These pro-inflammatory mediators disrupt colonic mucosal integrity and compromise intestinal immune homeostasis. Similarly, aberrant activation of the MAPK signaling cascade in UC promotes excessive cytokine release through phosphorylation of downstream signaling proteins, thereby exacerbating disease progression [44,46]. Previous studies have demonstrated that natural polysaccharides, such as those derived from *Mesona chinensis* Benth and *Zingiber officinale*, can suppress these pathways, thereby attenuating cytokine overproduction and inflammatory responses [47,48]. In the present study, CYP treatment inhibited the activation of both the MAPK and NF-κB signaling pathways by reducing the phosphorylation levels of key regulatory proteins. These findings are consistent with earlier reports on the anti-inflammatory activity of polysaccharides. Notably, alterations in NF-κB- and MAPK signaling-related proteins were retained under microbiota-depleted conditions.

The gut microbiota is widely considered an important determinant of UC pathogenesis [49,50]. However, not all polysaccharides act only through microbiota modulation. Polysaccharides derived from *Atractylodes macrocephala* Koidz. have demonstrated ameliorative effects in UC through the concurrent modulation of the gut microbiota and host immune responses [51]. Non-digestible oligosaccharides can exert direct, microbiota-independent effects on immune cells in germ-free mice [52]. *Polygonatum cyrtonema* Hua fructan has been reported to ameliorate ulcerative colitis through both microbiota-dependent and microbiota-independent mechanisms [53]. Previous studies have reported that CYP can ameliorate colitis by modulating the gut microbiota [20,24]. In the present study, the retention of protective efficacy after antibiotic pretreatment suggests that an intact gut microbiota may not be absolutely required for CYP to exert protective effects. Natural polysaccharides exert anti-inflammatory effects through two principal mechanisms: one mechanism involves the direct modulation of immune and intestinal epithelial cells, whereby polysaccharides stimulate cell proliferation, enhance the secretion of immunoregulatory factors, and support epithelial function, thereby attenuating inflammatory responses; the other mechanism is mediated by the gut microbiota, which degrades polysaccharides into SCFAs that serve as the primary energy source for intestinal epithelial cells and help maintain intestinal barrier integrity [16,54,55]. These findings may suggest that CYP may modulate inflammatory responses, intestinal barrier function, and antioxidant-related parameters through potential direct effects on intestinal epithelial or immune cells, while microbial modulation may also contribute to the therapeutic effects when microbial integrity is preserved. Collectively, these findings indicate that host-associated regulatory effects may represent one component of polysaccharide-mediated protection in colitis.

## 5. Conclusions

In conclusion, CYP alleviated DSS-induced UC by attenuating inflammation, improving epithelial barrier function, and enhancing antioxidant defenses. Notably, its protective efficacy was preserved under conditions of severe gut microbiota suppression, suggesting that its therapeutic effects may not be exclusively dependent on microbiota modulation. These findings highlight the therapeutic potential of CYP in colitis and suggest that its effects may involve direct actions on immune and intestinal epithelial cells, supporting further exploration of its role as a complementary therapeutic agent in IBD.

## Data Availability

The original contributions presented in this study are included in the article/Supplementary Materials. Further inquiries can be directed to the corresponding authors.

## Supplementary Materials

The following supporting information can be downloaded at https://www.mdpi.com/article/10.3390/foods15101633/s1. Figure S1: Picture of colon length; Figure S2: Comparative heatmap visualization of functional pathway abundances across KEGG (A), MetaCyc (B), and COG (C) databases; Table S1: Disease activity index score; Table S2: Histopathology score.
