# Supplementary material for: Chinese Yam Polysaccharide Alleviates DSS-Induced Ulcerative Colitis After Antibiotic Pretreatment
Source: Foods. 2026 May 8;15(10):1633. doi: 10.3390/foods15101633 (PMC13205214; doi:10.3390/foods15101633)
Supplement: Supplementary file 1 [file foods-15-01633-s001.zip › foods-4266748-supplementary.pdf]

Supplementary Materials

Figure S1

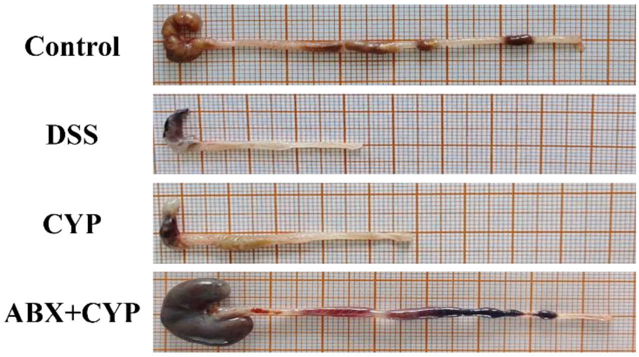

Figure S1. Picture of colon length.

Figure S2

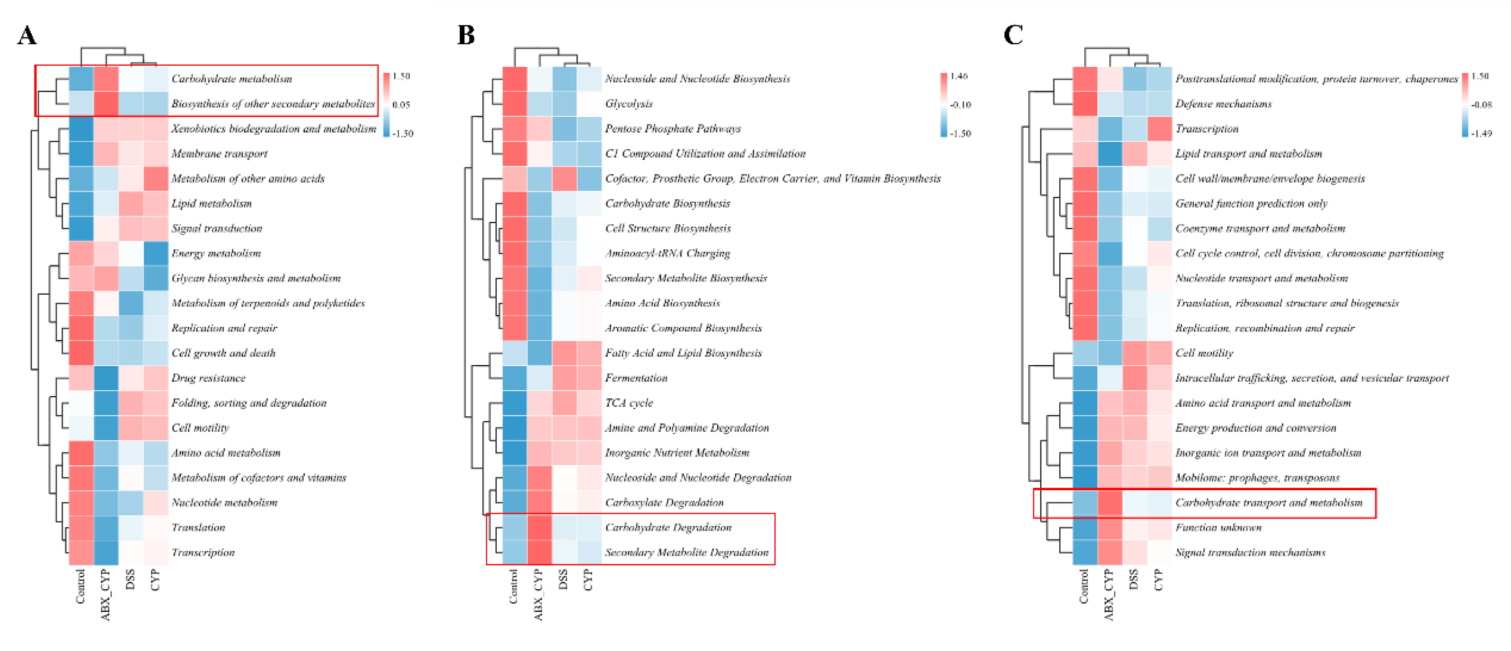

Figure S2. Comparative heatmap visualization of functional pathway abundances across KEGG (A), MetaCyc (B), and COG (C) databases.

Table S1. Disease activity index score.

Body weight loss	Feces consistency	Degree of hematochezia	Score
< 1%	normalcy	normalcy	0
1-5%	Shaped but loose	slight hemoccult	1
5-10%	Soft stool	Stool with blood stains	2
10-15%	Mucoid stool	Visible in stool	3
>15%	Watery stool	Gross bleeding	4

Table S2. Histopathology score.

Extent of lesions	Crypt injury rate	Invasion depth	Inflammation	Score
normal	normal	normal	normal	0
1-25%	1-25%	mucosal	minimal	1
25-50%	25-50%	mucosal and submucosal	mild	2
50-75%	50-75%	penetration of muscularis propria	moderate	3
>75%	>75%	full thickness involvement	severe	4
